# Management of Parkinson’s disease challenged by co-morbid drug abuse

**DOI:** 10.1192/j.eurpsy.2022.1188

**Published:** 2022-09-01

**Authors:** J. Simas, A. Delgado, R. Guedes, C. Silveira, C. Soares

**Affiliations:** 1 Centro Hospitalar do Tâmega e Sousa, Department Of Psychiatry And Mental Health, Penafiel, Portugal; 2 Unidade Local de Saúde de Matosinhos, Department Of Mental Health, Matosinhos, Portugal; 3 Centro Hospitalar de Leiria, Department Of Mental Health And Psychiatry, Leiria, Portugal; 4 Centro Hospitalar Universitário de São João, EPE, Department Of Psychiatry, Porto, Portugal; 5 Medical Faculty of Porto University, Department Of Clinic Neurosciences And Mental Health, Porto, Portugal; 6 Centro Hospitalar Universitário de São João, EPE, Department Of Neurology, Porto, Portugal

**Keywords:** Addiction, Drug Abuse, Neuropsychiatry, Parkinson’s Disease

## Abstract

**Introduction:**

Parkinson’s disease (PD) is a neurologic degenerative condition with complex neuropsychiatric manifestations which can be challenging to manage and greatly impact quality of life and prognosis.

**Objectives:**

The description of this case aims to highlight the complex interaction between PD, drug-abuse and impulse control disorder (ICD).

**Methods:**

Clinical information was obtained through patient interviewing and medical records consulting. A literature review on the topic was conducted.

**Results:**

We report the case of a 52-years-old male with PD diagnosed at the age of 45, presenting with rigidity of right limbs and freezing of gait. He had a history of multiple substance-abuse: hashish, heroin and cocaine, with cessation of all substances by the age of 40. The patient responded well to antiparkinsonian medication initially, but needed frequent adjustments and developed ICD secondary to dopamine agonists, presenting pathological gambling and hypersexuality. At 47 he restarted using cocaine stating that it diminished the motor symptoms. Motor symptoms worsened and became partially responsive to medication. Pharmacologic options were limited due to ICD. He developed dopamine dysregulation syndrome, abusing dopaminergic drugs and requesting multiple prescriptions. Deep brain stimulation surgery was proposed, but the patient was deemed unfit for the procedure after two separate psychiatric evaluations, mainly because of behaviour and social problems in relation to sustained cocaine abuse and personality disorder. Attempts to stop drug abuse were unsuccessful despite several interventions by addiction psychiatry.

**Conclusions:**

Co-occurrence of PD, substance-abuse and personality disorder poses as a therapeutic challenge conditioned by pharmacological iatrogenesis and behavioural disturbances, requiring a multidisciplinary and individualized approach.

**Disclosure:**

No significant relationships.

